# The Psychiatric Risk Gene *Transcription Factor 4* (*TCF4*) Regulates Neurodevelopmental Pathways Associated With Schizophrenia, Autism, and Intellectual Disability

**DOI:** 10.1093/schbul/sbx164

**Published:** 2017-12-08

**Authors:** Marc P Forrest, Matthew J Hill, David H Kavanagh, Katherine E Tansey, Adrian J Waite, Derek J Blake

**Affiliations:** 1 Division of Psychological Medicine and Clinical Neurosciences, MRC Centre for Neuropsychiatric Genetics and Genomics, School of Medicine, Cardiff University, UK; 2 College of Biomedical and Life Sciences, Cardiff University, Cardiff, UK

**Keywords:** transcription factor, gene expression, schizophrenia, psychiatric genetics, neurodevelopment, functional genomics

## Abstract

**Background:**

Common genetic variants in and around the gene encoding transcription factor 4 (*TCF4*) are associated with an increased risk of schizophrenia. Conversely, rare damaging *TCF4* mutations cause Pitt–Hopkins syndrome and have also been found in individuals with intellectual disability (ID) and autism spectrum disorder (ASD).

**Methods:**

Chromatin immunoprecipitation and next generation sequencing were used to identify the genomic targets of TCF4. These data were integrated with expression, epigenetic and disease gene sets using a range of computational tools.

**Results:**

We identify 10604 TCF4 binding sites in the genome that were assigned to 5437 genes. De novo motif enrichment found that most TCF4 binding sites contained at least one E-box (5′-CAtcTG). Approximately 77% of TCF4 binding sites overlapped with the H3K27ac histone modification for active enhancers. Enrichment analysis on the set of TCF4 targets identified numerous, highly significant functional clusters for pathways including nervous system development, ion transport and signal transduction, and co-expression modules for genes associated with synaptic function and brain development. Importantly, we found that genes harboring de novo mutations in schizophrenia (*P* = 5.3 × 10^−7^), ASD (*P* = 2.5 × 10^−4^), and ID (*P* = 7.6 × 10^−3^) were also enriched among TCF4 targets. TCF4 binding sites were also found at other schizophrenia risk loci including the nicotinic acetylcholine receptor cluster, *CHRNA5*/*CHRNA3*/*CHRNB4* and *SETD1A*.

**Conclusions:**

These data demonstrate that TCF4 binding sites are found in a large number of neuronal genes that include many genetic risk factors for common neurodevelopmental disorders.

## Introduction

Genetic variants in and around the transcription factor 4 (*TCF4*) gene are associated with range of disorders that are frequently associated with cognitive dysfunction.^1–3^ The most recent schizophrenia GWAS reported three independent single nucleotide polymorphisms (SNPs) in *TCF4* that surpassed the threshold for genome wide significance.^4^ Intriguingly, rare *TCF4* single nucleotide variants (SNVs) have also been described in schizophrenia patients, although their impact on the function of the protein has not been established.^5,6^ In addition to the genetic studies in schizophrenia, *TCF4* variants are associated with early information processing and cognitive markers, some of which are schizophrenia endophenotypes.^7–10^ Damaging *TCF4* mutations have also been described in large-scale genotyping studies in patients with ID, neurodevelopmental disorders, and most recently ASD.^11–15^ Haploinsufficiency of *TCF4* causes Pitt–Hopkins syndrome (PTHS); a rare form intellectual disability (ID) associated with characteristic facial features, autonomic dysfunction, and behavioral traits that resemble autism spectrum disorder (ASD).^16–19^ Collectively, these studies implicate TCF4 in a range of neurodevelopmental disorders.

TCF4 is a member of the basic helix-loop-helix (bHLH) family of proteins.^20–22^ For the purposes of disambiguation, it should be noted that *TCF4* (Gene ID: 6925) described herein should not be confused with T-cell factor 4 (Gene ID: 6934, official gene symbol, *TCF7L2*) since they can share the same acronym. TCF4 and its paralogues, collectively known as E-proteins, interact with other bHLH proteins to regulate DNA binding specificity and transcriptional activity at promoters and enhancers that contain E-boxes (5′-CANNTG).^2,20,23^ The human *TCF4* gene encodes multiple protein isoforms of which only the major isoforms TCF4-A and TCF4-B have been characterized in detail.^24^ In neurons, TCF4 regulates the intrinsic excitability of pyramidal cells of the prefrontal cortex and has been shown to attenuate neurite branching.^25,26^ Furthermore, haploinsufficiency of *Tcf4* in mice affects gene expression and DNA methylation in the brain, leading to enhanced long-term potentiation, learning and memory deficits, and autistic-like behavior.^22,25,26^ By contrast, mice over-expressing *Tcf4* in the brain display deficits in sensorimotor gating, fear conditioning, and circadian processes as well as impairments in attentional and behavioral anticipation.^7,27^

Although the function of TCF4 as transcription factor has been well established, very little is known about the genes regulated by TCF4 in neuronal cells and specifically in cells of human origin. Therefore, to gain a functional insight into the role of TCF4 in schizophrenia other neurodevelopmental disorders, we used a bespoke TCF4 antibody for chromatin immunoprecipitation and next generation sequencing (ChIP-seq) to define the genomic targets of TCF4. We found that TCF4 target genes cluster in neurodevelopmental pathways and are enriched for schizophrenia, ASD, and ID risk genes.

## Materials and Methods

Detailed methods are provided in the supplementary material.

### TCF4 Antibodies and Constructs

Rabbit polyclonal antibodies were raised against amino acids 361–554 (spanning TCF4-A and -B) of human TCF4-B fused to thioredoxin (TCF4_01) or glutathione S-transferase (TCF4_02), as described previously.^28^ Affinity purified antisera were tested extensively for specificity and reactivity by western blotting, immunoprecipitation, and mass spectrometry following ENCODE’s guidelines (supplementary figure S1).^29,30^ TCF4 expression constructs were used as described previously while the myc-E47 construct was kindly provided by Dr Carme Gallego.^31^

### Chromatin Immunoprecipitation and Next Generation Sequencing

ChIP-seq was performed on TCF4-enriched chromatin prepared from human SH-SY5Y neuroblastoma cells following ENCODE’s guidelines.^29,30^ Enriched chromatin was processed and sequenced by Source BioScience using the Illumina TruSeq ChIP Sample Preparation Kit. Reads were aligned to the human reference genome (UCSC/h19) with BWA (version 0.7.5a) using the MEM algorithm with default parameters.^32^ Peaks were called according to the ENCODE irreproducible discovery rate (IDR) analysis pipeline (https://sites.google.com/site/anshulkundaje/projects/idr). These data are available from the Gene Expression Omnibus (GEO) database with the accession number GSE96915 (http://www.ncbi.nlm.nih.gov/geo/query/acc.cgi?acc=GSE96915). The HOMER ChIP-seq analysis package^33^ was used to annotate peaks, link them to nearest transcription start site and for motif analysis. All identified peaks were considered for annotation and analysis.

### Gene Set Enrichment Analysis

Gene set/pathway enrichment analysis was performed using DAVID.^34,35^ All genes annotated with a TCF4 peak were considered and the whole genome used as background. For analyses using gene expression data, only those genes present on the microarray were used as background. Enrichment analysis using custom genes sets was performed using the Fisher’s exact test implemented in STATA 12.0 or *R* using the appropriate background lists (eg, genes detected on a microarray, protein coding genes, etc.) cited in each publication. Lists of genes containing de novo variants identified in patients and controls were obtained from Fromer et al.^36^ FMRP targets were obtained from Darnell et al,^37^ whereas the list of loss of function (LoF) intolerant human genes was obtained from Lek et al.^38^ Further details of these custom gene sets are provided in the supplementary material. Enrichment of TCF4 target genes at schizophrenia risk loci was investigated using the multi-marker analysis of genomic annotation (MAGMA) package using a 10 kb window as described previously.^39,40^

## Results

### Identification of Genomic TCF4 Binding Sites by ChIP-seq

To perform chromatin immunoprecipitation (ChIP) experiments we generated TCF4 polyclonal antibodies (TCF4_01 and TCF4_02) that would detect the 2 major TCF4 isoforms, TCF4-A and TCF4-B. Antibodies were tested for their specificity and ability to immunoprecipitate TCF4 in SH-SY5Y cells following the ENCODE guidelines.^29^ SH-SY5Y neuroblastoma cells are one of the most widely used cell models to study neurodevelopmental processes and have been extensively used for neuropsychiatric research. TCF4_01 specifically detected over-expressed and endogenous TCF4 in SH-SY5Y cells but did not detect the TCF4 paralogue E47 (TCF3) (supplementary figure S1A). Mass spectrometry confirmed that TCF4_01 and TCF4_02 immunoprecipitated endogenous TCF4-A and TCF4-B isoforms from SH-SY5Y cells (supplementary figures S1B and S1C).

**Fig. 1.**
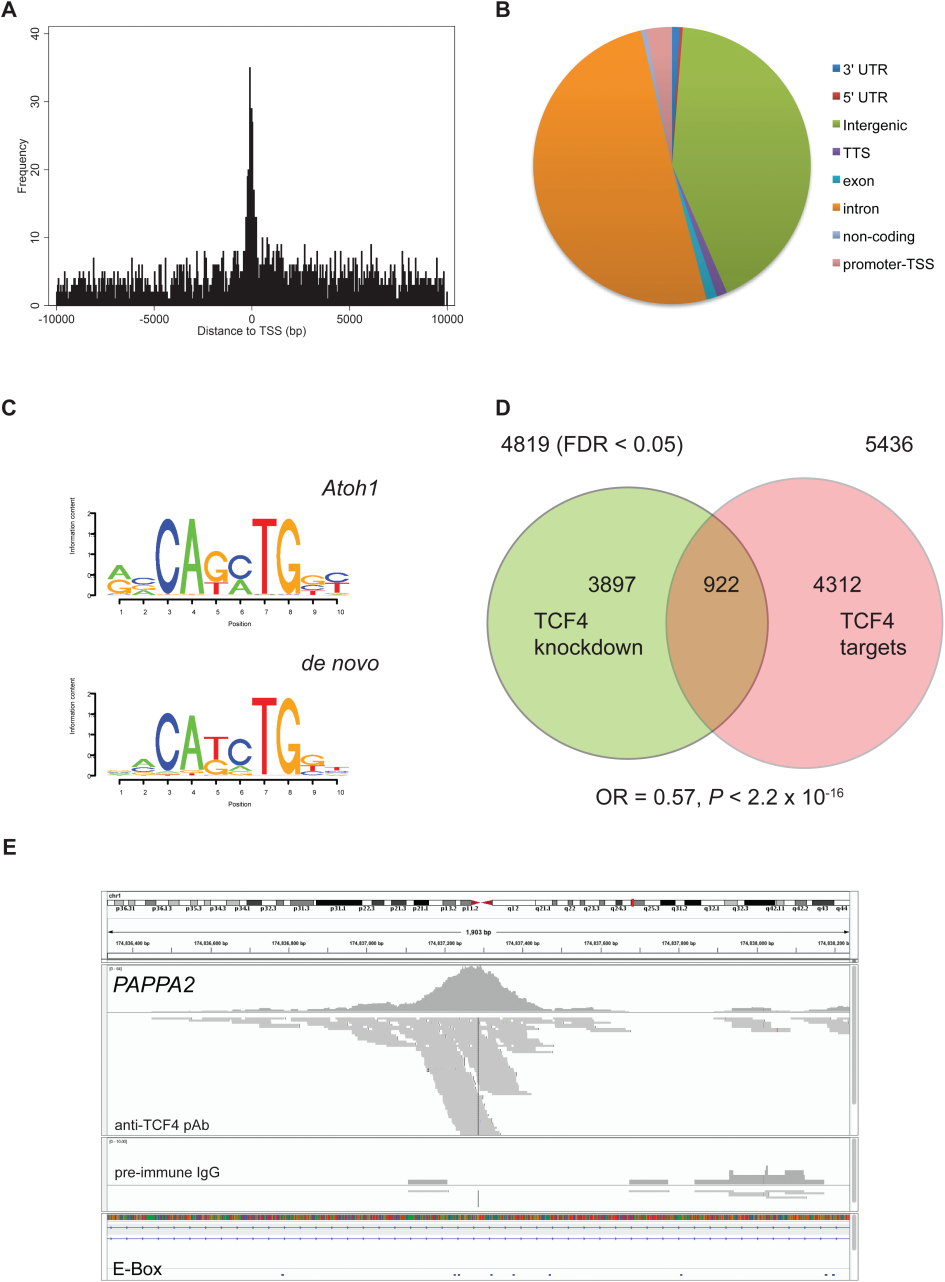
Chromatin immunoprecipitation and next generation sequencing (ChIP-seq) analysis of genomic transcription factor 4 (TCF4) binding sites. Genomic distribution of TCF4 ChIP-seq peaks centered around the transcriptional start site (A). Location of TCF4 binding sites relative to genomic features (B). TCF4 consensus binding sites predicted from the motif discovery program in HOMER (C). TCF4 binding sites are enriched at canonical E-boxes (Atoh1), while de novo motif discovery identified a nonpalindromic E-box. Venn diagram showing the overlap between TCF4 targets and differentially expressed genes in TCF4-depleted cells (D). Statistical tests were performed against a background set of 16318 expressed genes with TORAY 3D-Gene and HOMER annotations. TCF4 bound regions at *PAPPA2* displayed using IGV (E). IGV plots for *PAPPA2* (intron) showing tiled, unique sequence reads after PCR de-duplication. For comparative purposes, the control pre-immune IgG is also shown. The locations of canonical E-boxes within the locus are also shown.

High throughput sequencing of TCF4 and IgG ChIP samples produced greater than 2.2 × 10^7^ reads per sample with Phred quality scores (>Q30) of 98.34% or higher. MACS 2.0 detected 10604 high confidence peaks, spanning 0.18% of the genome, from duplicate samples that passed IDR analysis. These peaks are distributed around transcriptional start sites (TSSs) but are located primarily in intronic or intergenic regions of the genome (figures 1A and 1B). Only 4.3% of peaks occur in promoter regions immediately 5′ of the TSS (figure 1B).

DNA sequence motif analysis of TCF4 bound regions of the genome using HOMER identified strong enrichment of the Atoh1 (proneural atonal gene) E-box motif (*P* = 1 × 10^−1003^) compared to the whole genome. De novo motif construction also confirmed the enrichment of an E-box motif in these peaks, albeit with a slightly different base composition (figure 1B). Implementing both the canonical and de novo E-box motif, we annotated each ChIP-seq peak with the number of E-boxes occurring within a 200 bp window of the peak maximum. 85.8% of all peaks contained at least one canonical E-box, with the most frequent count being 4, ie, 2 separate sites with a count given to each strand. A similar pattern was also observed for the nonpalindromic de novo motif.

### TCF4 Binding Sites and Differentially Expressed Genes in TCF4-Depleted Cells

Each of the 10604 peaks was mapped to the nearest TSS, resulting in 5436 unique target genes. Of these unique genes, 4218 were annotated as protein coding. To examine the relationship between TCF4 binding sites and gene expression, we compared the list of TCF4 target genes with those that were differentially expressed in TCF4-depleted SH-SY5Y cells.^41^ Of the 4819 differentially expressed genes, only 922 (17.6%) contained TCF4 binding sites (figure 1D). To search for functional TCF4 binding sites as defined by Cusanovich et al,^42^ we restricted our analysis to TCF4 bound regions of the genome within 10 kb of a TSS. Of the 1310 genes that had TCF4 binding sites within 10kb of the TSS, 234 (17.9%) were differentially expressed in TCF4-depleted cells. Similarly, genes with a TCF4 binding site within 10 kb of the TSS were also under-represented among the differentially expressed genes.

TCF4 bound genes were visualized using the Integrative Genomic Viewer (IGV) (figure 1E and supplementary figure S2).^43^Figure 1E shows a representative example of a TCF4 bound region at an intronic site within *PAPPA2* (chr1:176570227-176570965). TCF4 binding sites are centered around densely tiled sequence reads spanning approximately 750 bp of chromosome 1 in the TCF4 ChIP track (anti-TCF4 pAb) encompassing 5 canonical E-boxes (figure 1E). Importantly, these peaks are absent from the IgG track where affinity purified pre-immune IgG was used as the immunoprecipitating antibody. *PAPPA2* is also down-regulated (−1.95-fold) in TCF4-depleted cells (figure 1E).^41^ Quantitative PCR confirmed that TCF4_01 enriched the TCF4 bound region of *PAPPA2* (35.8-fold) compared to pre-immune IgG.

### TCF4 Binding Sites Are Associated With Active Enhancers

**Fig. 2.**
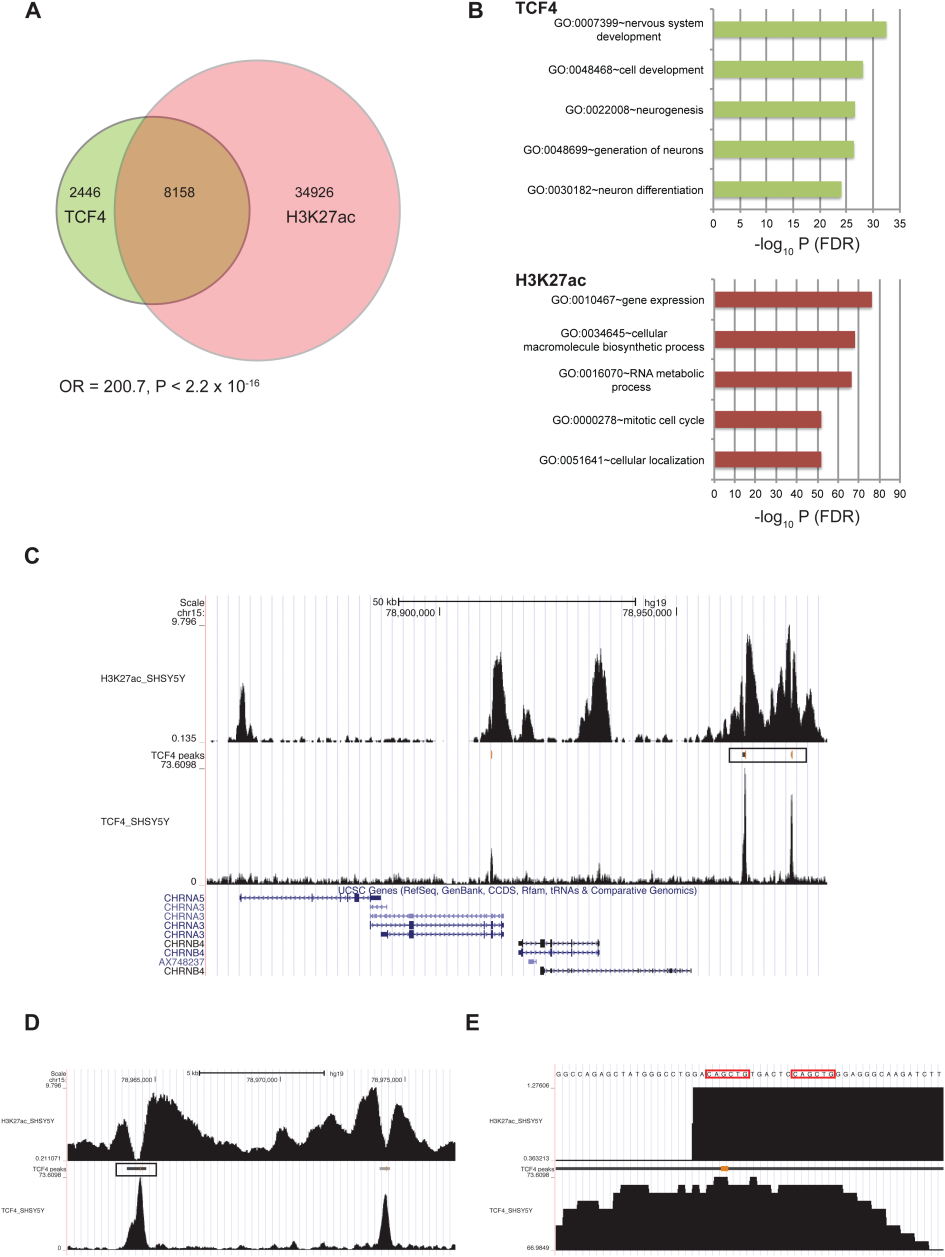
Transcription factor 4 (TCF4) binding sites are enriched at active enhancers. Venn diagram showing the intersection of TCF4 bound regions of genome with those marked with the H3K27ac in SH-SY5Ycells (A). Top ranked GO terms for TCF4 bound genes and genes marked by H3K27ac in SH-SY5Y cells (B). UCSC genome browser screenshot of the *CHRNA5/A3/B4* locus on human chromosome 15 showing ChIP-seq peaks for H3K27ac and TCF4 (C). The 2 major TCF4 peaks (box) are found at an active enhancer (marked by extensive H3K27ac) upstream of *CHRNB4*. Enlarged view of panel C showing that TCF4 bound regions are located within histone-depleted regions (troughs) of the enhancer (D). Enlarged view of panel C showing the TCF4 peak maps to a pair of E-boxes (highlighted) with the sequence 5′-CAGCTG (E).

To further annotate TCF4 binding sites to regulatory regions of the genome, we used BEDtools to compare the distribution of TCF4 bound regions of the genome with the histone modifications, histone H3 lysine 27 acetylation (H3K27ac, active enhancers), histone H3 lysine 4 monomethylation (H3K4me1, enhancers), and histone H3 lysine 4 trimethylation (H3K4me3, promoters) in SH-SY5Y cells. Intersecting the locations of the TCF4 bound regions of the genome with those marked by H3K27ac revealed that 76.9% of the TCF4 bound regions of the genome overlap the H3K27ac histone mark (*P* < 2.2 × 10^−16^, OR = 200.7, figure 2A). Comparison of the ontologies of TCF4 bound genes with those marked by H3K27ac showed that neurodevelopment processes were the top gene ontology (GO) terms for TCF4 (see below) whereas the top ontologies for H3K27ac were gene expression and cell cycle (figure 2B). Similarly, we examined the association of TCF4 bound regions of the genome with those marked by H3K4me1 and H3K4me3. In common with H3K27ac, 77.0% of TCF4 peaks were marked by H3K4me1 (*P* < 2.2 × 10^−16^, OR = 201.8). By contrast, only 1.67% of TCF4 peaks were marked by H3K4me3 (*P* < 2.2 × 10^−16^, OR = 3.86) in accordance with the low percentage of TCF4 binding sites at the promoter-TSS region of genes (figure 1B).

### TCF4 Regulates Gene Expression at the CHRNA5/CHRNA3/CHRNB4 Locus

We further examined the relationship between TCF4 bound regions of the genome and histone modifications at specific loci; focusing on TCF4 binding sites that mapped to common variant schizophrenia risk loci discovered by the Psychiatric Genomics Consortium (PGC2).^4^ We observed 4 TCF4 binding sites that mapped to the *CHRNA5*/*CHRNA3*/*CHRNB4* locus that encodes subunits of the nicotinic acetylcholine receptor (nAChR) on chromosome 15 (figure 2C). Two TCF4 binding sites (chr15:78963775–78964722 and chr15: 78973914–78974467) are located in an enhancer region marked by extensive H3K27ac. The 2 TCF4 peaks in this region interdigitate with the troughs of H3K27ac demarcating nucleosome-depleted regions of the genome that associated with dense transcription factor occupancy (figure 2D).^44^ At higher resolution, maximal sequence coverage spans 2 E-boxes located within the H3K27ac trough (figure 2E). Importantly, *CHRNA3* and *CHRNB4* are down-regulated (−1.30 and −1.35-fold, respectively) following knockdown of TCF4 in SH-SY5Y cells.^41^ These data suggest that TCF4 may regulate gene expression at the *CHRNA5*/*CHRNA3*/*CHRNB4* locus demonstrating a functional association between two distinct schizophrenia risk genes/loci.

### Biological Processes Associated With TCF4 Bound Genes

GO analysis on the 5436 unique target genes revealed highly significant enrichments of many gene categories. Five hundred thirty-six terms were significant at a *q* value <0.05 of which 312 were unique terms. The most significant term was “GO:0007399~nervous system development” (figure 2B). Given the large number of significant terms we used EnrichmentMap to group and visualize these 312 terms.^45^ Nine clusters containing 6 or more terms were considered for further analysis (figure 2 and supplementary figure S3). Each cluster relates to distinct biology and we manually assigned descriptions based on the terms within each cluster. The 3 largest clusters relate to cation channel function, neurogenesis, development and morphology, and signaling (figure 2 and supplementary figure S3). The remaining clusters include terms relating to apoptosis and mesenchymal cell differentiation (supplementary figure S3) as identified previously.^41^

### TCF4 Binding Sites Are Enriched in Neurodevelopmental Genes

To determine the physiological relevance of TCF4 bound genes in SH-SY5Y cells to gene expression in the human brain, we examined the enrichment of the complete list of TCF4 targets in a number of commonly used gene sets.^37,38,46^ Following the rationale that genome-wide co-expression networks reflect biological processes essential to human neocortical development we mapped TCF4 targets onto co-expression modules described by Parikshak et al.^46^ TCF4 bound genes were enriched for modules 17 and 18 but were significantly under-represented in modules 4, 6, 12, and 14 (table 1). Module 17 contains genes involved in synaptic transmission and cation channel activity and is also enriched for ASD genes (table 1).^46^ Similarly, we also undertook gene set analysis on the list of targets recognized by the FMRP RNA-binding protein and LoF intolerant genes. The targets of the FMRP-RNA binding protein and LoF intolerant genes are enriched for genes that harbor both de novo and common and risk alleles for schizophrenia and other neurodevelopmental disorders such as ASD and ID.^4,36,47,48^ TCF4 bound genes were also highly enriched for FMRP targets and LoF intolerant genes (OR = 1.87 and 1.42, respectively, *P* < 2.2 × 10^−16^) (table 1). Taken together these data suggest that TCF4 binds to genes in SH-SY5Y cells that are representative of neuronal gene expression pathways that regulate important neurodevelopmental cellular processes in human brain.

**Table 1.** Gene Set Enrichment for TCF4 Targets in Cortical Expression Modules, FMRP Targets, and LoF Intolerant Genes

Module	Ontology^a^	*P* value	*P* Corr.	OR	95% CI
17	Synaptic transmission	8.34 × 10^−8^	1.50 × 10^−6^	1.55	1.32–1.81
18	Defense response	2.15 × 10^−7^	3.87 × 10^−6^	1.54	1.32–1.81
13	Synaptic transmission	3.77 × 10^−3^	NS	1.29	1.09–1.53
8	Negative regulation of neuron differentiation	1.26 × 10^−1^	NS	1.29	0.94–1.76
1	None	7.82 × 10^−3^	NS	1.29	1.07–1.54
11	Cell cycle	3.43 × 10^−1^	NS	1.11	0.89–1.37
15	Response to virus	4.94 × 10^−1^	NS	1.10	0.84–1.43
5	None	3.35 × 10^−1^	NS	1.09	0.91–1.30
16	Cation-transporting ATPase activity	6.48 × 10^−1^	NS	1.06	0.85–1.32
2	Zinc ion binding	9.28 × 10^−1^	NS	0.99	0.82–1.18
None	None	2.66 × 10^−1^	NS	0.96	0.89–1.03
3	Nucleic acid metabolic processes	1.42 × 10^−1^	NS	0.88	0.74–1.04
9	Regulation of cellular amino acid metabolic process	4.46 × 10^−2^	NS	0.69	0.48–0.99
10	None	3.40 × 10^−1^	NS	0.62	0.26–1.49
6	None	8.76 × 10^−5^	1.58 × 10^−3^	0.54	0.39–0.75
12	None	7.28 × 10^−6^	1.31 × 10^−4^	0.52	0.39–0.71
14	Translational elongation	4.15 × 10^−9^	7.47 × 10^−8^	0.48	0.37–0.63
4	None	4.78 × 10^−7^	8.61 × 10^−6^	0.45	0.32–0.63
Gene set					
FMRP	Synaptic transmission	<2.2 × 10^−16^		1.87	1.62–2.14
LoF	Regulation of transcription	<2.2 × 10^−16^		1.42	1.31–1.53

*Note*: Gene sets were collated from Parikshak et al (weighted gene co-expression network, human neocortical development), Darnell et al (FMRP bound genes) and Lek et al (LoF intolerant genes).^37,38,46^ Initial *P* values were generated using Fisher’s exact test and were corrected (*P* corr) for multiple testing (Bonferroni) where appropriate. Only *P* values <.05 were considered statistically significant. OR, odds ratio; CI, confidence interval; NS, not significant.

^a^Prinicipal ontology for each module or gene set.

### TCF4 Targets and Neuropsychiatric Disease Risk Genes

Given the association of *TCF4* variants with schizophrenia and other neurodevelopmental disorders, we examined the enrichment of TCF4 targets with genes associated with different diseases. Firstly, we determined the overlap of TCF4 targets with loci associated with schizophrenia through common genetic variation.^4^ Forty-nine schizophrenia risk loci (45.4%) contained TCF4 binding sites out of 108 GWAS significant schizophrenia risk loci. A total of 4168 genes were present in both the TCF4 target and schizophrenia risk loci datasets. We used MAGMA to test for association between TCF4 bindings sites and schizophrenia (PGC2 GWAS) correcting for gene length and linkage disequilibrium (LD).^39^ MAGMA reported a nominally significant enrichment of TCF4 target genes amongst schizophrenia risk loci (*P* = .049) after correcting for gene length and LD.

Although there was only nominal enrichment of TCF4 targets in the GWAS significant schizophrenia risk loci, several schizophrenia risk genes were found to contain TCF4 binding sites. As described above, the *CHRNA5/A3/B4* locus contained several TCF4-binding sites in regulatory regions including a distal enhancer (figure 2C). Other schizophrenia risk loci with TCF4 binding sites include *DRD2*, *TSNARE1*, and *GRIA1*, all of which are down-regulated in TCF4-depleted cells and *MIR137/DPYD*. Furthermore, several TCF4 binding sites are also located within the *TCF4* gene itself suggesting that TCF4 may autoregulate the expression of its numerous distinct isoforms.^24^ In addition to the common variant schizophrenia risk loci, rare LoF mutations in *SETD1A* have recently been associated with schizophrenia and developmental disorders.^49,50^*SETD1A*, that encodes a histone methyltransferase, has a TCF4 binding site (chr16:30968306–30969367) at its TSS, potentially implicating both schizophrenia risk genes in the same regulatory network.

Next, we determined the overlap between TCF4 bound genes and de novo variants found in individuals with schizophrenia collated by Fromer et al.^36^ For schizophrenia de novos, statistically significant enrichment was observed for all classes of mutations bar silent mutations (table 2); all mutations (SCZ_all *P* = 5.25 × 10^−7^), loss of function mutations (SCZ_LoF *P* = 2.79 × 10^−2^) and nonsynonymous mutations (SCZ_NS, *P* = 1.26 × 10^−5^). Importantly, no enrichment was observed in the control datasets derived from unaffected siblings and controls irrespective of mutation type. Finally, we examined the overlap between TCF4 bound genes and loci implicated in ID and ASD through de novo mutations (table 2). Significant enrichment also was detected in both ID and ASD for all mutations types (ID_all *P* = 7.61 × 10^−3^; ASD_all *P* = 2.46 × 10^−4^) and for nonsynonymous mutations (ID_NS *P* = 2.04 × 10^−3^; ASD_NS *P* = 1.57 × 10^−4^). Silent mutations and the most damaging LoF mutations were not enriched in either ID or ASD de novos.

**Table 2.** Gene Set Enrichment for TCF4 Bound Genes and Neuropsychiatric Disease Risk Genes

Gene Set	Gene Count	*P* Value	*P* Corr.	OR	95% CI
SCZ_all	862	3.28 × 10^−8^	5.25 × 10^−7^	1.55	1.33–1.81
SCZ_LoF	98	1.74 × 10^−3^	2.79 × 10^−2^	1.99	1.31–3.03
SCZ_NS	668	7.90 × 10^−7^	1.26 × 10^−5^	1.56	1.31–1.85
SCZ_silent	207	1.02 × 10^−2^	NS	1.5	1.11–2.03
ASD_all	951	1.54 × 10^−5^	2.46 × 10^−4^	1.39	1.20–1.62
ASD_LoF	124	3.54 × 10^−2^	NS	1.53	1.04–2.27
ASD_NS	724	9.84 × 10^−6^	1.57 × 10^−4^	1.47	1.25–1.74
ASD_silent	248	2.40 × 10^−1^	NS	1.20	0.89–1.60
ID_all	154	4.76 × 10^−4^	7.61 × 10^−3^	1.86	1.33–2.61
ID_LoF	30	2.60 × 10^−1^	NS	1.6	0.73–3.51
ID_NS	129	1.28 × 10^−4^	2.04 × 10^−3^	2.09	1.45–2.99
ID_silent	25	1.00	NS	0.94	0.35–2.49
Control/sibling_all	557	8.39 × 10^−3^	NS	1.3	1.07–1.58
Control/sibling_LoF	49	3.80 × 10^−1^	NS	1.35	0.72–2.55
Control/sibling_NS	417	5.98 × 10^−2^	NS	1.25	1.00–1.56
Control/sibling_silent	151	7.15 × 10^−2^	NS	1.4	0.98–2.00

*Note*: Statistical enrichment for TCF4 bound genes in gene sets derived from exome sequencing studies in schizophrenia, ID and ASD using gene sets were collated from Fromer et al.^36^ Initial *P* values were generated using Fisher’s exact test and were corrected (*P* corr.) for multiple testing (Bonferroni). Only *P* values <.05 were considered statistically significant. OR, odds ratio; CI, confidence interval; NS, not significant.

## Discussion

The transcription factor TCF4 has been implicated in the genetic aetiology of several neuropsychiatric and neurological disorders, however, the identity of TCF4 target genes in neuronal cells remains largely unknown. In this study, we define the genome-wide TCF4 binding sites in the commonly used SH-SY5Y neuroblastoma cell line. As predicted from earlier in vitro studies, we found that the majority of TCF4 binding sites (85.8%) in the genome contained at least one canonical E-box within 200 bp of the peak maximum.^23^ Furthermore, de novo motif discovery showed that TCF4 binding sites were enriched for the nonpalindromic E-box sequence, 5′-CAtcTG (figure 1C). Interestingly, 77% of the TCF4 binding sites overlapped active enhancers marked by H3K27ac and H3K4me1 in SH-SY5Y cells (figure 2). Conversely, only 1.7% of TCF4 peaks were marked by H3K4me3 in agreement with the low percentage of TCF4 binding sites at the promoter-TSS region of genes (figure 1B). Taken together, these data demonstrate that TCF4 is an E-box-binding transcription factor that preferentially associates with active enhancers in SH-SY5Y cells.

**Fig. 3.**
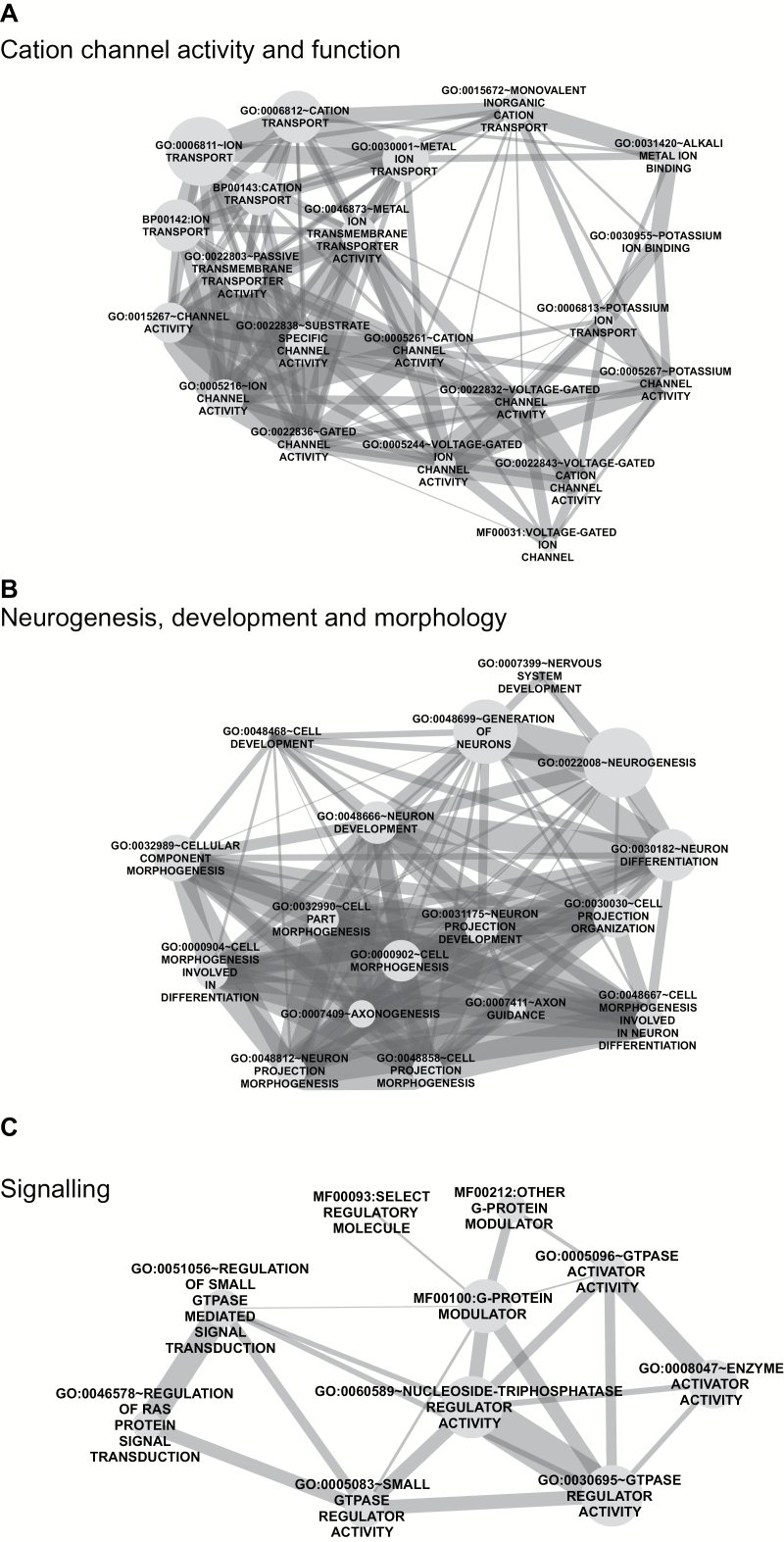
Networks of gene ontology (GO) terms derived from transcription factor 4 target genes. Nodes (circles) GO terms with the size proportional to the total number of member. The networks were produced using Cytoscape with connecting lines representing the degree of overlap between nodes. Clustered terms were manually cropped for visualization. Clusters correspond to the three most significant terms; cation channel activity and function (A), neurogenesis, development, and morphology (B), and signaling (C).

To understand how TCF4 binding modulates gene expression, we intersected the list of ChIP-defined targets with the list of differentially expressed genes following acute TCF4 knockdown in SH-SY5Y cells.^41^ Differentially expressed genes were significantly under-represented among the set of TCF4 targets (figure 1D). Although this finding seems counterintuitive, large scale studies transcriptomic studies have found that functional transcription factor binding occurs at only a small subset of genes.^42^ Furthermore, several differentially expressed TCF4 targets such as *NEUROG2*, *SNAI2*, and *DACH1* are transcriptional regulators whose altered expression will also contribute to transcriptional dysregulation in TCF4-depeleted cells. Given the large number of TCF4 bound genes identified, we used enrichment analysis to group biological processes involving TCF4 targets *en masse*. Consistent with a neurodevelopmental role for TCF4, TCF4’s genomic targets cluster into ontologies associated with a wide variety of cellular functions including nervous system development and signal transduction (figures 2B and 3; supplementary figure 3).

One of the principal aims of this study was to determine whether TCF4 binds and regulates genes implicated in the aetiology of complex neuropsychiatric disorders. Given the association of *TCF4* common variants with schizophrenia, we initially focused on TCF4 binding sites in schizophrenia risk loci identified in the most comprehensive genome wide association study published to date.^4^ Although there was only nominal enrichment of TCF4 targets among the genome-wide significant schizophrenia loci, many schizophrenia susceptibility loci contain functional TCF4 binding sites such as the *CHRNA5/A3/B4* locus which encodes subunits of the nAChR. In addition to schizophrenia, the *CHRNA5/A3/B4* locus is a risk factor for nicotine dependence, smoking behavior, and lung cancer.^51,52^*CHRNA3* polymorphisms are associated with sensorimotor gating, measured be prepulse inhibition (PPI), in schizophrenia patients and controls.^53^ Interestingly, nicotine administration essentially normalized the effects *CHRNA3* genotype on PPI suggesting that nicotine may interact with *CHRNA3* variants to modulate sensorimotor gating.^54^ Similarly, schizophrenia-associated *TCF4* risk variants also influence sensorimotor gating and interact with smoking behavior to modulate this neurophysiological response.^8,9^ Our finding that TCF4 directly regulates transcription of the *CHRNA5/A3/B4* cluster may provide a mechanistic explanation for the interaction between *TCF4* common variants with smoking behavior on auditory sensorimotor gating; considered to be an endophenotype of schizophrenia. Clearly, further research will be required to unravel the functional effects of *cis*-acting variants at the *CHRNA5/A3/B4* locus with TCF4 and other transcriptional regulators in schizophrenia and other neurodevelopmental disorders.

In addition to schizophrenia, we found a significant enrichment for TCF4 targets associated with genes implicated in ASD and to a lesser extent ID (table 2). We also observed similar convergence between TCF4 targets and ASD risk genes represented in co-expression modules during cortical development in humans (table 1). Notably, module 17 that contain genes involved in synaptic transmission are enriched for both inherited, common variant ASD-risk genes and TCF4 targets.^46^ Furthermore, a robust overlap exists between targets of the FMRP RNA-binding protein and genes implicated in a range of brain disorders including schizophrenia and ASD.^36,55^ We found that many TCF4 bound genes are also FMRP targets, including TCF4 itself (table 1). The overlap between FMRP targets and TCF4-bound regions of the genome suggests that both genes may operate in a similar pathway. It is clear that TCF4 and FMRP, the protein product of the fragile X syndrome ID gene, have regulatory functions in neurons that are essential for normal cognitive development and synaptic plasticity.^25,56^ Furthermore, FMRP and TCF4 are regulated by neuronal activity suggesting that their activities may be co-regulated.^57,58^ Finally, protein truncating mutations in a subset of LoF intolerant genes are frequently found in individuals with ASD ND ID/developmental delay (DD).^59^ The enrichment of TCF4 targets among LoF intolerant genes lends further support to a convergent role for TCF4 regulated gene expression networks in mediating elements of the disease risk mechanism in a range of neurodevelopmental disorders (table 1).

In conclusion, the identification of genome-wide binding sites for TCF4 provides an insight into the biological processes regulated by TCF4 in human cells. The identification of a large repertoire of TCF4 regulated genes in cells of neuronal origin will provide a useful adjunct for the interpretation of regulatory processes in the brain. Applying this information to common psychiatric disorders may provide a mechanistic insight into the shared genetic aetiology between different diagnoses, yielding functional regulatory interactions among candidate risk genes.

## Supplementary Material

Supplementary data are available at *Schizophrenia Bulletin* online.

## Funding

This work was funded by the Medical Research Council (MRC, MR/L010305/1). M.P.F. was funded by a MRC PhD studentship and an MRC Centenary Award. M.J.H. was supported by a Neuroscience and Mental Health Research Institute, Cardiff University Fellowship.

## Supplementary Material

Supplementary-Material
