# Supplementary material for: The Psychiatric Risk Gene Transcription Factor 4 (TCF4) Regulates Neurodevelopmental Pathways Associated With Schizophrenia, Autism, and Intellectual Disability
Source: Schizophr Bull. 2017 Dec 8;44(5):1100–10. doi: 10.1093/schbul/sbx164 (PMC6101561; doi:10.1093/schbul/sbx164)
Supplement: Supplementary-Material [file sbx164_suppl_supplementary-material.doc]

**The psychiatric risk gene *Transcription Factor 4* (*TCF4*) regulates neurodevelopmental pathways associated with schizophrenia, autism and intellectual disability**

**Forrest *et al*.**

**Supplementary Material**

**Supplementary Methods**

*Chromatin immunoprecipitation*

SH-SY5Y cells were cultured in Dulbecco’s modified Eagle medium (Thermo Fisher Scientific, Waltham MA, USA) supplemented with 10% (*v/v*) foetal calf serum (GE Healthcare Life Sciences, Little Chalfont, UK) without antibiotics. Confluent cells were fixed with 1% (*v/v*) formaldehyde for 5min at room temperature and then quenched with 125mM glycine. Cells were then washed twice with cold phosphate buffered saline (PBS) and collected by scraping into cold PBS. Cells were pelleted by centrifugation (2000g, 5min, 4°C) and then washed in PBS and collected by centrifugation. 1 x 10^7^ pelleted cells were lysed in 1ml ChIP buffer (150mM NaCl, 50mM Tris-HCl (pH 7.5), 5mM EDTA, NP-40 (0.5% *v/v*), Triton X-100 (1.0% *v/v*) for 30min on ice. Nuclei were released by passing the lysate through a 23-gauge needle. Nuclei were collected by centrifugation (2000g, 5min, 4°C), the supernatant was discarded and remaining pellet resuspended in 1ml of shearing buffer (50mM Tris pH 8.0, 5mM EDTA, 150mM NaCl, 0.1% (*v/v*) SDS). The lysate was then clarified by centrifugation (13,000rpm for 10min at 4°C) and the supernatant transferred to a new tube. Chromatin was fragmented by sonication using a Covaris S2 (Covaris, Woburn MA, USA) following the manufacturer’s recommendations. Following sonication, SDS was sequestered by addition of Triton X-100 to a final concentration of 1% (*v/v*). Sonicated chromatin was then pre-cleared for 1h at 4°C using bovine serum albumin (BSA) blocked beads.

For each ChIP reaction, chromatin prepared from approximately 1 x 10^7^ cells was incubated with 5µg anti-TCF4 or anti-IgG antibody, overnight at 4°C with rotation. The chromatin was then centrifuged (13,000rpm for 10min at 4°C) and the supernatant transferred to a tube containing BSA-blocked beads. Protein-antibody complexes were then captured for 30min at 4°C with rotation. Beads were then washed twice with ChIP buffer containing 0.5M NaCl, followed by five washed with LiCl wash buffer (50mM Tris pH 8.0, 5mM EDTA, 0.5% (*v/v*) NP-40, 1.0% (*v/v*) Triton X-100, 500mM LiCl and then twice with rinse buffer (50mM Tris pH 8.0, 5mM EDTA). Beads were then resuspended in 100µl of elution buffer (10mM Tris pH 8.0, 1mM EDTA, 0.1% (*w/v*) SDS) and incubated with RNase for 30 min at 37°C). Proteinase K was added and incubated over night at 60°C. The eluate was then cleaned up using the QIAgen PCR clean up Kit (Qiagen, Valencia CA, USA).

*Peak calling and sequence analysis*

Peaks were called using MACS2 ^1^ in each replicate against a pooled IgG negative control sample using a *P* value cut-off of 0.05. The replicates were then randomly split into pseudo replicates and peaks called in those using the same parameters. Finally, peaks were called in the pooled replicates and in random pseudo replicates of the pooled data. IDR analysis suggested the optimum number of peaks from the pooled replicates was 10,604. These peaks were used in all subsequent analyses. The Integrative Genomics Viewer IGV (version 2.3.38) was used for peak visualisation. The complete TCF4 ChIP data set is available from the Gene Expression Omnibus (GEO) database with the accession number GSE96915 (<http://www.ncbi.nlm.nih.gov/geo/query/acc.cgi?acc=GSE96915)>. ChIP-seq data for the histone acetylation mark in SH-SY5Y cells were obtained from the GEO database as follows;

H3K27ac (<https://www.ncbi.nlm.nih.gov/geo/query/acc.cgi?acc=GSE65664>) ^2^, H3K4me1 (<https://www.ncbi.nlm.nih.gov/geo/query/acc.cgi?acc=GSM1532409>) ^3^ and H3K4me3 (<https://www.ncbi.nlm.nih.gov/geo/query/acc.cgi?acc=GSM518558)> ^4^. Browser extensible data (bed) files were manipulated and intersected using the BEDtools software package ^5^.

*Quantitative PCR (qPCR)*

The following primer pairs (forward and reverse) were used to quantitate the enrichment of TCF4 bound regions of the genome in the TCF4 ChIP over IgG ChIP.

*PAPPA2*, 5’-GCTATGGCAGCACAAAACAA and 5’-TCTGGGTTGCAGATGTTTGA; *CHRNB4*, 5’-CCCAGAAACAGGACTTGGAA and 5’-ACAGGACTCCCTGAGACGAG; *DPT*, 5’-TGGTGACTAGGGGCAGAAGT and 5’-TGGATCCTTCTCACCCACAT; *RNU5F-1*, 5’-TGTGTCACATTTGCCCTCAT and 5’-TGCAGATATCGGCTCAAGTG; *LINC00398*, 5’-GTGCCAAGCCTCCAAGAAG and 5’-CCATGCTCTGCATATGGTGT; *OPRD1*, 5’-CCTGCAGGACAGATGGAGAT and 5’-CAGGGAGGAATGGAAAATCA; *SYPL1*, 5’-CACTGTGGCTCCATTGTCTG and 5’-AAGCAGGGCTTTGTGTCACT.

Prior to PCR, IGV plots (supplementary figure S2) were used to refine TCF4 bound regions associated with the *PAPPA2, DPT, CHRNB4, SYPL1, ORPD1, LINC00398* and *RNU5F-1* genes. For each gene, TCF4_01 enriched the target region over the IgG control (data not shown). In control experiments, genomic regions lacking TCF4 binding sites were not enriched in TCF4 bound chromatin compared to control IgG.

*Gene Set Enrichment Analysis*

The following gene set categories were used for all analyses: GO biological process (levels 4, 5 and FAT), GO cellular component (levels 4, 5 and FAT), GO molecular function (levels 4, 5 and FAT), KEGG pathways, OMIM, Panther pathways and Panther biological processes. Grouping and visualisation of gene set relationships was performed using the Cytoscape plugin EnrichmentMap ^6^. Gene categories with a FDR < 0.05 were considered and grouped by overlapping genes with a Jaccard coefficient of > 0.4. Groups with six or more nodes were considered for further commentary. In addition to the GO enrichment, several custom expression and disease gene sets were interrogated (see below). The set of differentially expressed genes in TCF4-depleted SH-SY5Y cells was obtained from Forrest *et al*., (<https://www.ncbi.nlm.nih.gov/geo/query/acc.cgi?acc=GSE48367>) ^7^. Co-expression modules for neocortical development were obtained from Parikshak *et al.*, ^8^ where a background set of 15,885 genes for used for comparison. Similarly, a list of FMRP targets was obtained from Darnell *et al.*, ^9^ and enrichment analysis was restricted to human paralogues of the protein coding genes using a background list of 20,651 protein coding genes. Enrichment of TCF4 target genes at schizophrenia risk loci was investigated using the multi-marker analysis of genomic annotation (MAGMA) package using a 10kb window as described previously ^10, 11^. Summary results from the Psychiatric Genomics Consortium’s study of schizophrenia (PGC2 data set) were obtained from <https://www.med.unc.edu/pgc/files/resultfiles/scz2.snp.results.txt.gz>. Variants within the MHC region (chr6:26Mb-34Mb) were removed. MAGMA was run with default settings using a gene start/stop extension of 10kb. Lists of genes containing *de novo* variants identified in patients and controls were obtained from Fromer *et al*. ^12^ which collated data from Iossifov *et al*., ^13^; Neale *et al*., ^14^; O’Roak *et al*., ^15^; Sanders *et al*., ^16^; Rauch *et al*. ^17^; Xu *et al*., ^18^; de Ligt *et al*., ^19^; Girard *et al*., ^20^; Gulsuner *et al*., ^21^. For enrichment analysis, each group (all, loss of function, non-synonymous and silent) was defined according to Fromer *et al*., and TCF4 ChIP target genes were restricted to those annotated as protein coding using a background set of 20,651 genes ^12^. A list of loss of function (LoF) intolerant human genes was obtained from Lek *et al*., ^22^ where a background list of 18,226 genes was used to test for enrichment.

**Additional References**

**1.** Zhang Y, Liu T, Meyer CA, et al. Model-based analysis of ChIP-Seq (MACS). *Genome Biol.* 2008;9:R137.

**2.** Oldridge DA, Wood AC, Weichert-Leahey N, et al. Genetic predisposition to neuroblastoma mediated by a LMO1 super-enhancer polymorphism. *Nature.* 2015;528:418-421.

**3.** Chipumuro E, Marco E, Christensen CL, et al. CDK7 inhibition suppresses super-enhancer-linked oncogenic transcription in MYCN-driven cancer. *Cell.* 2014;159:1126-1139.

**4.** Kleine-Kohlbrecher D, Christensen J, Vandamme J, et al. A functional link between the histone demethylase PHF8 and the transcription factor ZNF711 in X-linked mental retardation. *Mol Cell.* 2010;38:165-178.

**5.** Quinlan AR, Hall IM. BEDTools: a flexible suite of utilities for comparing genomic features. *Bioinformatics.* 2010;26:841-842.

**6.** Merico D, Isserlin R, Stueker O, Emili A, Bader GD. Enrichment map: a network-based method for gene-set enrichment visualization and interpretation. *PLoS One.* 2010;5:e13984.

**7.** Forrest MP, Waite AJ, Martin-Rendon E, Blake DJ. Knockdown of human TCF4 affects multiple signaling pathways involved in cell survival, epithelial to mesenchymal transition and neuronal differentiation. *PLoS One.* 2013;8:e73169.

**8.** Parikshak NN, Luo R, Zhang A, Won H, Lowe JK, Chandran V, Horvath S, Geschwind DH. Integrative functional genomic analyses implicate specific molecular pathways and circuits in autism. *Cell.* 2013;155:1008-1021.

**9.** Darnell JC, Van Driesche SJ, Zhang C, et al. FMRP stalls ribosomal translocation on mRNAs linked to synaptic function and autism. *Cell.* 2011;146:247-261.

**10.** de Leeuw CA, Mooij JM, Heskes T, Posthuma D. MAGMA: generalized gene-set analysis of GWAS data. *PLoS Comput Biol.* 2015;11:e1004219.

**11.** Hill MJ, Killick R, Navarrete K, Maruszak A, McLaughlin GM, Williams BP, Bray NJ. Knockdown of the schizophrenia susceptibility gene TCF4 alters gene expression and proliferation of progenitor cells from the developing human neocortex. *J Psychiatry Neurosci.* 2016;41:160073.

**12.** Fromer M, Pocklington AJ, Kavanagh DH, et al. De novo mutations in schizophrenia implicate synaptic networks. *Nature.* 2014;506:179-184.

**13.** Iossifov I, Ronemus M, Levy D, et al. De novo gene disruptions in children on the autistic spectrum. *Neuron.* 2012;74:285-299.

**14.** Neale BM, Kou Y, Liu L, et al. Patterns and rates of exonic de novo mutations in autism spectrum disorders. *Nature.* 2012;485:242-245.

**15.** O'Roak BJ, Vives L, Girirajan S, et al. Sporadic autism exomes reveal a highly interconnected protein network of de novo mutations. *Nature.* 2012;485:246-250.

**16.** Sanders SJ, Murtha MT, Gupta AR, et al. De novo mutations revealed by whole-exome sequencing are strongly associated with autism. *Nature.* 2012;485:237-241.

**17.** Rauch A, Wieczorek D, Graf E, et al. Range of genetic mutations associated with severe non-syndromic sporadic intellectual disability: an exome sequencing study. *Lancet.* 2012;380:1674-1682.

**18.** Xu B, Ionita-Laza I, Roos JL, et al. De novo gene mutations highlight patterns of genetic and neural complexity in schizophrenia. *Nat Genet.* 2012;44:1365-1369.

**19.** de Ligt J, Willemsen MH, van Bon BW, et al. Diagnostic exome sequencing in persons with severe intellectual disability. *N Engl J Med.* 2012;367:1921-1929.

**20.** Girard SL, Gauthier J, Noreau A, et al. Increased exonic de novo mutation rate in individuals with schizophrenia. *Nat Genet.* 2011;43:860-863.

**21.** Gulsuner S, Walsh T, Watts AC, et al. Spatial and temporal mapping of de novo mutations in schizophrenia to a fetal prefrontal cortical network. *Cell.* 2013;154:518-529.

**22.** Lek M, Karczewski KJ, Minikel EV, et al. Analysis of protein-coding genetic variation in 60,706 humans. *Nature.* 2016;536:285-291.

**Supplementary Figures**

**Supplementary Fig. S1** Characterization of anti-TCF4 antibodies used for ChIP.

**C**

Western blotting and mass spectrometry were used to determine the reactivity and specificity of the anti-TCF4 polyclonal antibodies. Affinity purified anti-TCF4 polyclonal TCF4_01 detect endogenous TCF4-A (55kDa) and TCF4-B (72kDa) in untransfected SH-SY5Y cells (**A**). Two channel imaging shows that TCF4_01 detects over-expressed, myc-tagged and GFP-tagged TCF4-B (red) but does not detect over-expressed myc-tagged or untagged E47 (green). TCF4_01 and TCF4_02 were used to immunoaffinity purify endogenous TCF4 from SH-SY5Y cells (**B**). Each antibody efficiently immunoprecipitates TCF4-A and TCF4-B from total protein extracts prepared in RIPA buffer. TCF4-A and TCF4-B are immunodepleted from RIPA extracts following immunoprecipitation. In control experiments, TCF4 isoforms were not immunoprecipitated with IgG or when the primary antibody was omitted. Mass spectrometry identification of TCF4 isoforms immunoprecipitated from SH-SY5Y cells (**C**). Peptides covering each TCF4 isoform are shown in red text. Peptides unique to TCF4-B (NH_2_-NGPTSLASGHFTGSNVEDR) and TCF4-A (NH_2_-MYCAYTIPGMGGNSLMYYYNGK) were readily identified.

**Supplementary Fig. S2** TCF4 binding sites in different genes displayed using the Integrative Genomics Viewer (IGV).

IGV plots (NCBI36/hg18 assembly) for *DPT* (TSS), *CHRNB4* (intergenic, distal enhancer), *SYPL1* (intergenic), *ORPD1* (intron), *LINC00398* (intergenic) and *RNU5F-1* (intergenic) showing unique sequence read depth after de-duplication. Note that ChIP enrichment for each TCF4 bound region shown in the figure was independently confirmed using qPCR. Quantitative PCR was used to independently determine the fold enrichment (TCF4_01/ pre-immune IgG) for TCF4 bound regions of *DPT* (10.4 fold), *CHRNB4* (357.7 fold), *SYPL1* (13.3 fold), *OPRD1* (149.6 fold), *LINC00398* (9450.8 fold) and *RNU5F-1* (5555.3 fold). For comparative purposes, TCF4 ChIP-seq sequence reads are shown above those for the control pre-immune IgG. Note that peak heights are scaled to 120 for TCF4 and 10 for the pre-immune IgG controls. The locations of canonical E-boxes within the locus are also shown.

**Supplementary Fig. S3** Additional networks of enriched GO terms derived from TCF4 target genes.

**
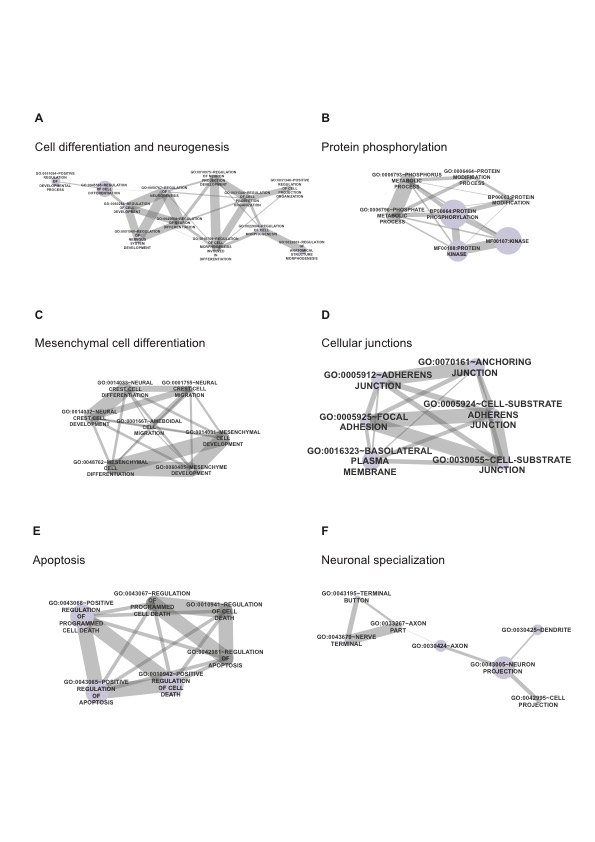
**

Nodes (circles) represent DAVID terms with the size proportional to the total number of member genes. The networks were produced using Cytoscape with connecting lines represent the degree of overlap between nodes. Clustered terms were manually cropped for visualization. Clusters correspond to; cell differentiation and neurogenesis (A), protein phosphorylation (B), mesenchymal cell differentiation (C), cellular junctions (D), apoptosis (E) and neuronal specialization (F).
